# Cognitive and Neuroimaging Correlates of the Insomnia Severity Index in Obstructive Sleep Apnea: A Pilot-Study

**DOI:** 10.3390/app11125314

**Published:** 2021-06-08

**Authors:** Alberto R. Ramos, Noam Alperin, Sang Lee, Kevin A. Gonzalez, Wassim Tarraf, Rene Hernandez-Cardenache

**Affiliations:** 1 Department of Neurology, Miller School of Medicine, University of Miami, Miami, FL 33136, USA; 2 Department of Radiology, Miller School of Medicine, University of Miami, Miami, FL 33136, USA;; 3 Department of Neuroscience, University of California, San Diego, CA 92093, USA;; 4 Department of Health Care Sciences, Institute of Gerontology, Wayne State University, Detroit, MI 48202, USA;; 5 Department of Psychiatry, Miller School of Medicine, University of Miami, Miami, FL 33136, USA;

**Keywords:** sleep apnea, hypoxemia, cognitive, brain health, MRI

## Abstract

We aim to determine the sleep correlates of age-related brain loss in a sample of middle-aged to older males with obstructive sleep apnea (OSA). We recruited consecutive treatment naïve male patients with moderate to severe OSA from January to November of 2019. We excluded participants if they had dementia, stroke or heart disease. We collected demographic variables and vascular risk factors. We also obtained the insomnia severity index, the Epworth sleepiness scale and the Pittsburgh sleep quality index. We also obtained computerized neurocognitive testing with the go-no-go response inhibition test, Stroop interference test, catch game test, staged information processing speed test, verbal memory test and non-verbal memory test. We derived age and education adjusted domain-specific *Z*-scores for global cognition, memory, attention, processing speed and executive function. We used brain MRI T1-weighted images to derive total hippocampal and gray matter volumes. Partial correlations evaluated associations between variables from sleep questionnaires (e.g., insomnia severity index score), and polysomnographic variables (the apnea-hypopnea index, average oxygen levels during sleep) with cognitive domains and brain volumes. We examined 16 participants with an age range of 40–76 years, 73% Hispanic/Latino. The mean apnea-hypopnea index was 48.9 ± 25.5 and average oxygen saturation during sleep was 91.4% ± 6.9%. Hypertension was seen in 66% and diabetes mellitus in 27%. We found that the insomnia severity index score and average oxygen levels during sleep had the strongest correlations with brain volumes and cognition. These preliminary findings may aid in developing future strategies to improve age-related brain loss in patients with OSA.

## 1. Introduction

Emerging research points to a critical role for sleep in cognition and the risk for Alzheimer’s disease (AD) [1]. Sleep disturbances [2–7], as well as, dental pathology, oral health [8–10] can accelerate the aging process and increase the risk for AD [6,11–15]. For example, obstructive sleep apnea (OSA), a common sleep disorder found in more than 25% of adults [16], is associated with cognitive decline and increased AD risk (HR 1.2 to 2.4) [17,18]. Obstructive sleep apnea leads to sleep fragmentation and disturbed sleep. Importantly, more than 50% of OSA patients may report difficulties falling or staying asleep [19]. Of interest, up to 38% of OSA patients meet the diagnostic criteria for having comorbid insomnia [19]. Patients with comorbid insomnia and OSA (COMISA) may be at increased risk for age-related cognitive decline and brain loss [19]. Therefore, we aimed to evaluate if the insomnia severity index was associated with cognitive and neuroimaging correlates of brain loss in OSA [20].

Structural brain characteristics may be indicative of neuronal injury and neurodegeneration that could precede the clinical deterioration commonly observed in AD. Recent use of advanced imaging techniques provides detailed information about the rate of brain tissue loss in specific brain regions associated with AD, such as the hippocampus [21,22]. There is limited data of symptom specific associations between the insomnia severity index and these markers of brain health in a sample of OSA patients. In this pilot-study we examined the feasibility of obtaining cognitive and neuroimaging markers of brain health in a diverse sample of middle-aged to older males from South Florida that include a large proportion of Hispanic/Latino adults. We assessed brain volumes and cognitive performance in newly diagnosed OSA patients [23]. For this analysis, we aimed to characterize the magnetic resonance imaging brain volumes and cognitive domains that correlate with the insomnia severity index in patients with OSA. More specifically, we tested the hypothesis that greater scores in the insomnia severity index, OSA severity (apnea-hypopnea index (AHI) and measures of nocturnal oxygenation (e.g., average oxygen levels during sleep) are associated with poorer cognitive function (global cognition, executive function, memory and processing speed) and greater global brain tissue loss in AD susceptible regions.

## 2. Materials and Methods

### 2.1. Population

We recruited consecutive patients referred to an academic sleep center from 1 January 2019 to 30 November 2019. The inclusion criteria were being male, ≥40 years of age with a new diagnosis of moderate to severe obstructive sleep apnea based on an apnea-hypopnea index of 15 events per hour of sleep, not previously treated for a sleep disorder. We excluded participants with chronic lung disease, history of stroke or transient ischemic attack, coronary artery disease, cardiac arrhythmia, cardiac failure or history of carotid/intracranial arterial stenosis. We also excluded participants with polysomnography defined central or complex sleep apnea [24], oxygen requirement, periodic limb movement disorder [25], referred for primary insomnia, use of sleep medication or benzodiazepines, obesity hypoventilation syndrome, or narcolepsy. In addition, we excluded participants unable to communicate verbally, or carried a diagnosis of dementia; current or prior history of neurological disease associated with cognitive impairment or with loss of gray or white matter, neurodevelopmental disorders, and claustrophobia. We obtained the cognitive and neuroimaging outcomes (explained below) during the same research visit. We instructed the participants to avoid caffeine, tobacco or medications that could interfere with the results of cognitive testing or brain magnetic resonance imaging. We used questionnaires and review of medical records to obtain demographic information (age, years of education, body mass index), health behaviors (tobacco, caffeine, alcohol use), medical history (diabetes mellitus, hypertension) and medication use. The study was approved by the Institutional Review Board at the University of Miami, Miller School of medicine. All participants signed an informed consent form.

### 2.2. Outcomes: Cognitive Function

We assessed cognitive function using a customized computerized-based cognitive assessment battery (NeuroTrax^™)^ [26] supervised by a licensed neuropsychologist or trained neuropsychometrician [27]. We assessed attention, executive functioning, verbal and visual memory, and speed of information processing [28]. We used the following subtests: the go-no-go response inhibition test, Stroop interference test, catch game test, staged information processing speed test, verbal memory test and non-verbal memory test [28]. The computerized scoring software calculated an overall global, composite and cognitive functioning score and the examiner assured completion of all parts of the assessment and reviewed all test findings. Final cognitive scores were normalized to age and years of education. The cognitive assessment also included validated depression [29,30] and anxiety scales [31,32].

### 2.3. Brain Magnetic Resonance Imaging (MRI)

All participants underwent MRI scanning using a 3T MRI scanner (skyra, Siemens Healthcare). Brain parcellation was obtained using a 3D T1-weighted sequence (MPRAGE) with 1.0 mm isotropic resolution, 2300 ms repetition time, 2.4 ms echo time, 930 ms inversion time, and 9-degree flip angle [33]. Total hippocampal volume was the sum of volumes of all subfields. In addition, the following regions were measured from the T1-weighted images, the total hippocampal volume, the intracranial volume (ICV), gray matter (GM), white matter (WM) and total brain tissue (i.e., GM + WM) volumes using FreeSurfer 5.3 (http://surfer.nmr.mgh.harvard.edu; accessed on 20 May 2020). All brain volumes were normalized to intracranial volume. The respiratory rate, oxygen saturation and heart rhythm were monitored during the MRI.

### 2.4. Main Exposures

#### 2.4.1. Sleep Symptoms

Participants completed the insomnia severity index (ISI), a 7-item instrument measuring the individual’s perception of insomnia symptoms [34]. The ISI is a widely used instrument which queries the presence or absence of insomnia syndrome (nocturnal and daytime symptoms) over the previous two weeks that has been validated in both English and Spanish [27]. We also obtained the Epworth sleepiness scale (ESS) [35], a widely-used tool with a validated Spanish version that assesses the likelihood of falling asleep in eight common situations. The Pittsburgh sleep quality index (PSQI) [36] which is a 19-item self-administered questionnaire with high internal consistency, test-retest reliability, diagnostic validity and a validated Spanish version. The questionnaires were completed in the participant’s preferred language (English or Spanish).

#### 2.4.2. Obstructive Sleep Apnea

We identified participants with obstructive sleep apnea that had in-laboratory video polysomnography (SandMan Elite), or a type-three home sleep apnea test (Embletta MPR). Overnight polysomnography used a standard sleep montage, including electroencephalographic, electromyography, and electrooculography monitoring for an in-laboratory overnight video-PSG. The PSG uses a finger pulse oximeter that calculates oxygen saturation and heart rate. The sleep stages, arousals, and sleep-related events and the apnea-hypopnea index, were recorded according to the established practice parameters of the American Academy of Sleep Medicine (AASM) [37]. Home sleep apnea studies used a self-applied type three device according to established AASM guidelines. A certified sleep technologist scored all records, manually edited artifacts, identified periods of sleep, and annotated each respiratory event using standardized techniques and the recommended scoring rules for apneas and hypopneas with its associated oxyhemoglobin desaturation. Moderate to severe OSA was defined with an apnea-hypopnea index ≥15 using the number of apneas and hypopneas/hour of sleep with 3% oxygen desaturation. We also obtained the average oxygen saturation during sleep, oxygen nadir during sleep and time spent with oxygen saturation in <90% (T90).

### 2.5. Statistical Analysis

All data was inspected to ensure values were within expected ranges. Descriptive statistics defined the mean, median and range across all continuous variables, while we used proportions for categorical variables. Pearson correlation was used to determine correlations between the insomnia severity index with demographic (age, years of education, body mass index), sleep variables, cognitive domains, neuroimaging, as well as depression and anxiety scales. We further adjusted for years of education (partial correlation) when evaluating the insomnia severity index and the apnea-hypopnea index with the cognitive domains and adjusted for age when evaluating the neuroimaging outcomes. All partial correlations adjusted for multiple testing. We evaluated correlations at *p* < 0.05 and *p* < 0.1.

In addition, we created an interactive dashboard to allow readers to visualize and interact with the data. The dashboard includes a (1) codebook of collected variables available for analyses, (2) a platform for generating descriptive statistics (e.g., means, medians and standard deviations) for all participants as well as by their linguistic preference (English and Spanish), (3) correlation plots to examine bivariate associations (correlations) between pairs of variables of interest; both linear and non-parametric fit lines can also be superimposed on the generated scatter plots and (4) partial correlations tables to examine adjusted associations between variables of interest; partial correlations are calculated using the “ppcor” package in R [38]. The dashboard was created using R software with the shiny package. The application can be accessed at https://sanarlab.shinyapps.io/Ramos_OSA_MRI/ (accessed on 6 February 2021).

## 3. Results

We recruited 16 male participants 40–76 years of age. Table 1 shows the demographic and main characteristics of the participants. The data from the sleep instruments indicated that our sample had a mean insomnia severity index of 10.3, reflecting sub-clinical insomnia on average. Five participants reported difficulties falling asleep, while eight participants reported difficulties staying sleep and four participants reported waking up too early.

The average Epworth sleepiness scale was normal, with a mean score of 8.2. Additionally, the average score of the Pittsburg sleep index was 5.4, reflecting poor sleep quality. Based on the items of the Pittsburgh sleep quality index four participants had difficulties falling asleep and 11 participants endorsed difficulties staying asleep or early awakening.

We observed a bivariate correlation between the insomnia severity index with the Epworth sleepiness scale, (r = 0.68, *p* = 0.0037), and with the average oxygen saturation (r = −0.53, *p* = 0.036).

The insomnia severity index had a negative correlation with attention score (r = −0.66, *p* = 0.015), adjusting for education and multiple comparison. The insomnia severity index did not correlate with other cognitive domains.

Of interest, the apnea-hypopnea index correlated with memory (r = −0.70, *p* = 0.01), while the Epworth sleepiness scale was negatively correlated with attention (r = −0.69, *p* = 0.02), adjusting for education and multiple comparisons. We also observed that average oxygen saturation during sleep had positive correlation with memory (r = 0.64), attention (r = 0.69) and global cognition (r = 0.57), but these were attenuated when adjusting for years of education. We also evaluated differences in verbal and visual memory subdomains and their relationships with the apnea-hypopnea index. We observed that visual memory had a statistically significant inverse correlation with apnea-hypopnea index (rho = −0.65, *p* < 0.01), but not with verbal memory functioning. The Pittsburgh sleep quality index did not correlate with the different cognitive domains.

### Correlations between the Insomnia Severity Index and Sleep Variables with Brain Volumes

After adjusting for age and multiple comparisons, the insomnia severity index had negative correlations with the caudal anterior cingulate cortex (r = 0.31, *p* = 0.04) and the inferior parietal gyrus (r = −0.75, *p* = 0.01); while the Epworth sleepiness scale correlated with the lateral ventricles (r = 0.83, *p* = 0.002). In addition, we observed age-adjusted partial correlations between average oxygen saturation during sleep and total cortical volume, lateral and medial orbitofrontal cortex, middle temporal cortex and precuneus, Table 2.

We did not observe correlations between the apnea-hypopnea index and the Pittsburgh sleep quality index with brain volumes. In addition, the insomnia severity index did not correlate with demographic (age, education, BMI), depression and anxiety scales.

## 4. Discussion

In this pilot-study of treatment naïve middle-aged to older males with moderate to severe OSA, we observed correlations between the insomnia severity index with decreased attention. In addition, we observed a positive correlation between the insomnia severity index with the anterior cingulate gyrus. Similar to our findings, patients with primary insomnia had increased brain volumes in the anterior cingulate gyrus [39], which has been associated with increased sleep-onset latency and wake after sleep onset [39], The anterior cingulate gyrus help process cognitive and emotional information [39], which could help further explain cognitive difficulties in OSA patients with possible comorbid insomnia. While most of our OSA patients had insomnia complaints, the small sample size and pilot nature of our study precluded us from disentangling nocturnal from daytime complaints, when using the insomnia severity index. Our findings could be explained by sleep fragmentation and associated sleep disruption in OSA. As suggested by others, future studies should isolate insomnia complaints in OSA patients, using the nocturnal sub-score of the insomnia severity index.

We did not observe a correlation between the apnea-hypopnea index and brain volumes. Multiple studies describe associations between the apnea-hypopnea index and either decreased or increased brain volumes [40]. The apnea-hypopnea index is the main diagnostic and treatment metric for OSA. Of interest, the average sleep oxygen saturation correlated with lower brain volumes. In a large population-based study, average oxygen saturation during sleep correlated with lower brain volumes in the hippocampus–amygdala complex and other cortical regions sensitive to low oxygen saturation [41]. These findings suggest that nocturnal oxygen saturation could be a better metric to study brain health in OSA patients. In addition, we observed that daytime sleepiness, as measured by the Epworth sleepiness scale, had positive correlations with the lateral ventricles, suggesting an increment in ventricular volume with increase daytime sleepiness. Human and animal studies suggest that daytime sleepiness could be the manifestation of hypoxemia-related brain damage [42–45], in OSA. Importantly, increased ventricular volumes was associated with a 46% increased dementia-risk in the “Atherosclerosis Risk in Communities Study” cohort [46].

Our findings implicate gross cerebral compromise, and more specifically, the potential impact to the medial temporal lobe, where both learning and general memory functions reside [47]. The correlations between the insomnia severity index and the Epworth sleepiness scale with cognitive and neuroimaging measures suggest a dysfunction of the frontal-temporal network.

Our findings suggest that advanced neuroimaging measures of brain health and cognition can serve as outcomes in OSA patients. Of importance, symptoms and metrics beyond the apnea-hypopnea index are necessary to identify at risk individuals, as well as to determine the interventions that could help reduce the impact of age-related cognitive decline and dementia in OSA patients.

The strengths of our pilot study include the systematic measurement of cognition and neuroimaging obtained during the same research day, along with validated sleep questionnaires. We also applied a stringent inclusion, exclusion criteria to maintain the internal validity of our study. Limitations include the small sample size, no comparison group, the cross-sectional design and limited adjustment for main confounders. The increased prevalence and severity of OSA in middle-aged males, coupled with better cognitive measures in females, limited us to examine males in our pilot study to avoid sex as a confounder. In our study, participants had either type three home sleep apnea studies or polysomnography. Compared to polysomnography home sleep apnea studies can underestimate the frequency of respiratory events, as well as preclude the assessment of sleep macro-or micro-architecture. However, type three home sleep apnea studies are an accepted method to diagnose OSA. Finally, our findings may not apply to females and need to be reproduce in a larger and heterogeneous sample.

In conclusion, we observed that the insomnia severity index, and average oxygen saturation during sleep, had strong correlations with cognitive and neuroimaging measures of brain health in middle-aged to older OSA patients. Findings that could help develop strategies to improve age-related brain loss in OSA.

## Figures and Tables

**Table 1.** Sample characteristics of treatment, naïve patients with moderate to severe sleep apnea.

Continuous Variables					
	Mean	SD	Median	Range	
Age, years	59.7	9.4	60.8	43.9	76.59
Years of education	16.4	3.9	16.5	6.0	22.0
Body mass index, kg/m^2^	29.9	4.5	29.4	22.4	36.4
Insomnia severity index	10.3	4.7	8.0	6.0	19.0
Epworth sleepiness scale	8.2	5.9	8.0	0.0	23.0
Pittsburgh sleep quality index	5.4	4.8	3.5	1.0	17.0
Depression score	2.5	2.9	2.0	0.0	10.0
Anxiety score	29.3	5.9	28.5	21.0	42.0
Apnea-hypopnea index	52.6	28.2	53.4	15.7	119.0
Average night-time oxygen saturation, %	91.4	6.9			
Oxygen saturation nadir, %	75.1	11.4			
Time with less than 90% oxygen saturation, minutes	32.9	35.6			
Categorical Variables					
Language	%	-	-	-	-
English	41%	-	-	-	-
Spanish	53%	-	-	-	-
Hispanic background	71%	-	-	-	-
Hypertension	59%	-	-	-	-
Diabetes Mellitus	24%	-	-	-	-
Coffee, cups per day		-	-	-	-
None	18%	-	-	-	-
1–2 cups	65%	-	-	-	-
3–5 cups	12%	-	-	-	-
Current tobacco	6%	-	-	-	-

**Table 2.** Correlations between insomnia, sleepiness and oxygen during sleep with brain volumes.

MRI Brain Volumes r (*p* Value)	Anterior Cingulate Cortex	Total Cortical Volume	Lateral Orbitofrontal Cortex	Medial Orbitofrontal Cortex	Middle Temporal Cortex	Precuneus	Inferior Parietal Gyrus
**Insomnia severity index**	0.31 (0.04)	-	-	-	-	-	−0.75 (0.01)
**Average oxygen saturation during sleep**	-	0.65 (0.06)	0.82 (0.003)	0.64 (0.07)	0.77 (0.01)	0.64 (0.07)	-

Partial correlations adjusted for age and multiple comparison.
